# Non-Invasive Monitoring of Cardiac Output in Critical Care Medicine

**DOI:** 10.3389/fmed.2017.00200

**Published:** 2017-11-20

**Authors:** Lee S. Nguyen, Pierre Squara

**Affiliations:** ^1^Critical Care Medicine Department, CMC Ambroise Paré, Neuilly-sur-Seine, France

**Keywords:** non-invasive monitoring, cardiac output, hemodynamics, critical care medicine, bioreactance

## Abstract

Critically ill patients require close hemodynamic monitoring to titrate treatment on a regular basis. It allows administering fluid with parsimony and adjusting inotropes and vasoactive drugs when necessary. Although invasive monitoring is considered as the reference method, non-invasive monitoring presents the obvious advantage of being associated with fewer complications, at the expanse of accuracy, precision, and step-response change. A great many methods and devices are now used over the world, and this article focuses on several of them, providing with a brief review of related underlying physical principles and validation articles analysis. Reviewed methods include electrical bioimpedance and bioreactance, respiratory-derived cardiac output (CO) monitoring technique, pulse wave transit time, ultrasound CO monitoring, multimodal algorithmic estimation, and inductance thoracocardiography. Quality criteria with which devices were reviewed included: accuracy (closeness of agreement between a measurement value and a true value of the measured), precision (closeness of agreement between replicate measurements on the same or similar objects under specified conditions), and step response change (delay between physiological change and its indication). Our conclusion is that the offer of non-invasive monitoring has improved in the past few years, even though further developments are needed to provide clinicians with sufficiently accurate devices for routine use, as alternative to invasive monitoring devices.

## Introduction

Hemodynamic instability requires cardiac output (CO) measurement and tracking to assess severity of disorders and to adjust treatments on a continuous basis. Invasive monitoring is widely used but is associated with inherent iatrogenic complications, notably for pulmonary catheters, esophageal probes, or arterial catheters (1–3). Therefore, non-invasive methods offer a safer approach even though their metrologic performance remains challenged, particularly in intensive care units (ICUs) (4, 5).

This article aims to review such non-invasive methods of CO monitoring excluding echographic, thermodilution, and pulse contour methods, already described in other sections. We will cover electrical bioimpedance and bioreactance, respiratory-derived CO monitoring technique, ultrasound CO monitoring, multimodal algorithmic estimation, and inductance thoracocardiography.

Devices are reviewed using three main metrologic criteria required for CO measurement: *trueness* (systematic error assessed by the closeness of agreement between the average of an infinite number of replicate measurements and the true or reference value), *precision* (random error assessed by the closeness of agreement between replicate measurements on the same or similar objects under specified conditions), and *step response change* (delay between physiological change and its indication) (6). Table 1 summarizes the metrologic performance of all reviewed technologies.

**Table 1:** Summarizes the metrologic performance of these different technologies.

Device	Author	Year	Number of patients	ICU setting	Mean bias (l/min)	Percentage error (%)	Precision (repeatability)
Bioimpedance	Peyton and Chong (69)	2010	435 (pooled)	Yes	−0.1 ± 1.1	Mild	nd
Bioreactance	Squara (20)	2007	110	Yes	+0.16 ± 0.52	Mild	12%
CO_2_ rebreathing	Kotake et al. (38)	2009	42	Yes	+0.18 ± 0.88	Mild	nd
	Peyton and Chong (69)	2010	167 (pooled)	Mixed	−0.05 ± 2.24	Mild	nd
	Opotowsky et al. (45)	2017	12232	Mixed	−0.4 ± 2.24	High	nd
Ultrasonic	Chong and Peyton (71)	2012	320 (pooled)	Yes	−0.39 ± 0.14	Poor	nd
Pulse wave velocity	Yamada et al. (51)	2012	213	Yes	+0.13 ± 1.15	Acceptable	nd
Inductance cardiography	Kaplan et al. (66)	2003	11	No	+0.2 ± 2.4	Mild	nd

## Bioimpedance and Bioreactance

**Figure 1:**
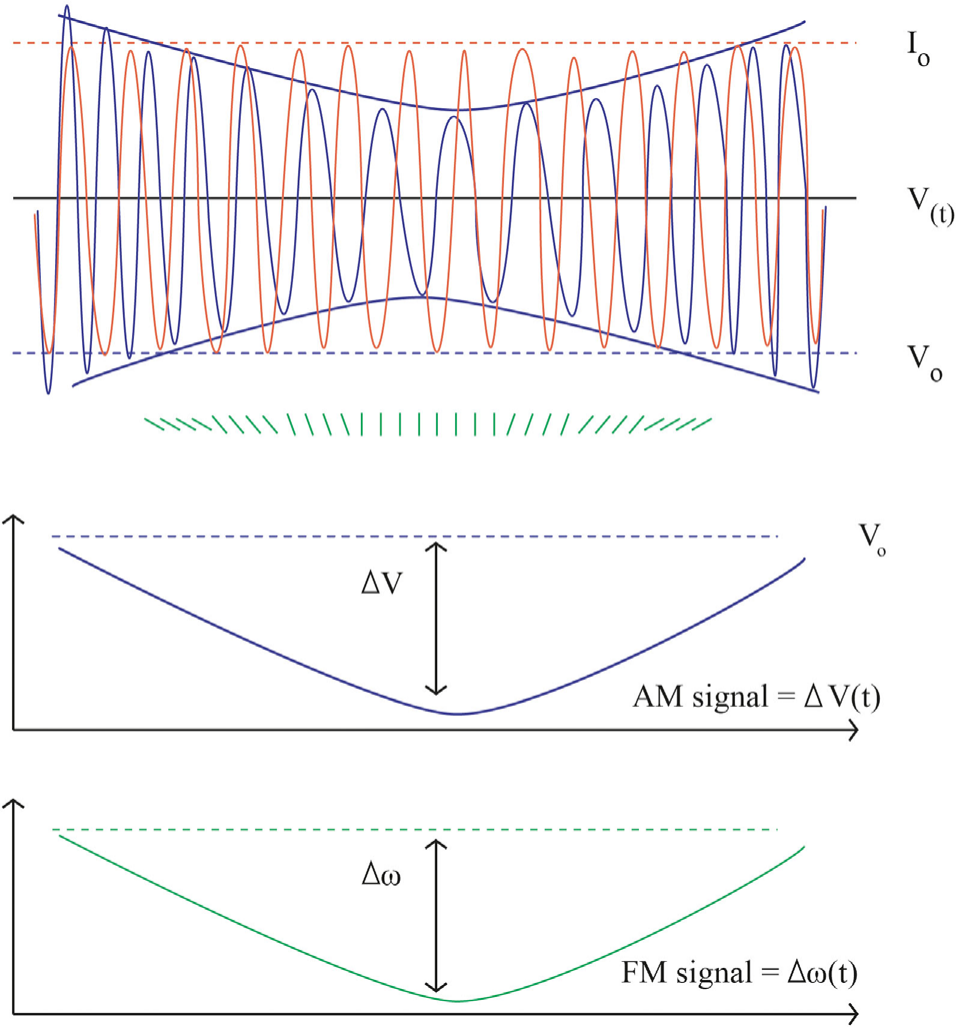
Bioimpedance and bioreactance signal. Upper part, in orange the input constant alternating current: *I*_o_ = 5 mA, frequency 75 kHz (ω = 150,000 radians/s). In blue the output voltage. *V*(*t*) = 200 ± 2 mV, frequency *F*(*t*) = 75 kHz ± 5 Hz. The instantaneous changes in phase are figured in green. In the middle, the *V*_o_ envelope (AM component) is extracted from the envelope of *V* = 4 mV, corresponding to the bioimpedance signal (*Z* = 4/5 = 0.8 Ω). The lower part shows the corresponding changes in frequency as obtained by the sum of instant phase shift (FM signal) figuring the bioreactance signal: *F* = 10 Hz (ω = 20 radians/s). Using appropriate scaling the shape of the AM and FM signals is the same.

Bioimpedance was first described in aeronautical medicine 50 years ago (7). It shares physical principles with bioreactance. It involves delivery of a low-amplitude high-frequency electrical current (*I*) across the thorax and received voltage (*V*) by electrodes. Hemodynamic variables: stroke volume (SV), CO, and thoracic fluid content (TFC) are then derived from the output signal fluctuation. Thoracic impedance (*Z*) is defined by the ratio *V*/*I*. At baseline (*Z*_o_) is the ratio of maximum values of *V* and *I* (*V*_o_/*I*_o_) and closely correlated changes in TFC (8–17). In the presence of flow through the aorta *Z*_0_ *Z* decreases over time proportionally to the increase of water and iron located in the chest, thus, to the increase in blood volume. Traditional bioimpedance systems use amplitude modulation as signal whereas bioreactance systems use frequency modulation and phase shifts (see Figure 1) (18). The theoretical superiority of the frequency modulation is its easier electric noise filtration (19).

A basic hypothesis to derive CO from both impedance and reactance is that the heart chambers are electrically isolated. Indeed, relatively to the chest with the lungs, the myocardial wall effectively provides electrical isolation to the content of the heart; therefore, changes in chest impedance and reactance are closely linked to variations of aortic volume. SV is obtained from the product of the ventricle ejection time and the slope of the initial change of the aortic volume obtained from the first derivative of the impedance or reactance signal (d*Z*/d*t*_max_ or d*X*/d*t*_max_). Since these changes only indicate relative changes of CO, a calibration factor (CF) is necessary, based on an initial cohort of patients to derive absolute values

$$\text{SV}=\text{VET}\times \text{d}Z/\text{d}t_{\text{max}}\times \text{ CF}$$

$$\text{SV = VET}\times \text{ d}X\text{/d}t_{\text{max}}\times \text{ CF}.$$

Several physical and anatomical hypotheses are required, limiting the effectiveness of impedance/reactance, most notably when there is no association between aortic systolic deformation and the SV (i.e., aortic dissection, aortic prosthesis), when hematocrit is very low, when pulmonary arterial pressure is elevated (for which, correction factors exist) or because of physical abnormalities such as obesity and dehydration (20).

Devices using bioimpedance include NCCOM (Bomed Medical, Irvine, CA, USA), BioZ (Cardiodynamics, San Diego, CA, USA), NICCOMO (MEDIS, Limenau, Germany), ICON (Osypka Cardiotronic, Berlin, Germany), ICG (Philips Medical Systems, Andover, MA, USA), NICOMON (Larsen and Toubro Ltd., Mumbai, India), the CSM3000 (Cheers Sails Medical, Shenzhen, China), and PHYSIOFLOW (Manatec Biomedical, Paris, France). The NICaS system (NI Medical, Petah-Tikva, Israel) uses the same principles but applied to the whole body. In the ECOM system (Ecom Medical, San Juan Capistrano, CA, USA), the transmitting and receiving electrodes are located on the cuff of an endotracheal tube, therefore close to the ascending aorta, in order to minimize the impact of analogous signals from other cardiac structures. Bioreactance is used by two products from the same company NICOM and Starling (Cheetah medical, Wilmington, DE, USA).

Bioimpedance and bioreactance have the strong advantage of being totally non-invasive and low costs. Literature on bioimpedance includes hundreds of articles, dozens of which are clinical trials set in a wide range of situations from ambulatory patients at home, to patients in a physiology laboratories, during surgery and in a ICU. Results are somewhat contradictory (21). At least a third of the publications failed to assess bioimpedance as a reliable mean to assess CO (22–25). Focusing on positive articles, most of them took place outside from an ICU setting most often in situations where the absolute value of CO has less importance than relative changes (26–30). This may be explained as electronical environment is heavier in ICU (due to the number of monitoring devices) compared to traditional medicine department; the higher the level of noise, the lesser bioimpedance would be accurate because of an unfavorable signal/noise ratio. Moreover, total body impedance is less accurate than localized thoracic impedance. Finally, even though last iterations of this technology seem more advanced (such as electrical velocimetry), results are not quite as clear either (31, 32). As of today, bioimpedance is not consensually viewed as accurate enough to estimate CO in ICU.

Bioreactance on the other hand has scarcer documentation. Theoretical superiority of bioreactance over bioimpedance was hinted in small sample studies set, in quite homogeneous patients of cardiac surgery ICU where the CF was derived (33, 34). In two studies, the accuracy, delay and amplitude of the signal were found similar to that of continuous thermodilution, although a bias up to 20% was found in 20% of patients. In other words, bioreactance-measured CO was similar to that of thermodilution in 80% of patients, but in those in whom it was not, bias could be as high as 20%. In several other studies investigating more heterogeneous patients, results were not considered as acceptable (35, 36). Concerns may be raised about decrease in accuracy during low-flow state and when electrocauterization was performed.

Further developments may be required to improve bioimpedance and bioreactance performance focusing or better understanding of the signal composition and better extraction of the aortic expansion signal. The auto calibration process may also be improved to fit better the studied population.

## Respiratory Derived CO Monitoring System: Partial CO_2_-Rebreathing

Applying Fick principles to exhaled gases allows measuring CO, by assessing oxygen consumption (VO_2_) and the difference of arterial (CaO_2_) and venous (CvO_2_) blood oxygen contents. This method was first described for intubated, sedated and ventilated patients (who did not present severe gas-exchange abnormality), using either oxygen (O_2_) or carbon dioxide (CO_2_) exhaled gas, and requires invasive arterial and mixed venous blood sampling, obeying the following equations (37):

$${\text{CO = VO}}_{2}{\text{/ CaO}}_{2}-{\text{CvO}}_{2}$$

$${\text{CO = VCO}}_{2}{\text{/ CaCO}}_{2}-{\text{CvCO}}_{2}.$$

A non-invasive method has since been developed, using the slope of CO_2_ dissociation curve (S) and the end tidal CO_2_ concentration (S. etCO_2_) as a surrogate of CaCO_2_. Since the CvCO_2_ is more difficult to estimate, it is derived considering two periods of time: normal respiration (*n*) and a 30-s period of rebreathing (*r*). Assuming that the CO and the CvCO_2_ remain unchanged during the two periods of time, the two equations become as follow:

$${\text{VO}}_{2}{\text{/ CaO}}_{2}-{\text{CvO}}_{2}\text{=}n{\text{VCO}}_{2}\text{/}n\left(\text{S}{\text{. etCO}}_{2}\right)-{\text{CvCO}}_{2}$$

$${\text{VCO}}_{2}{\text{/ CaCO}}_{2}-{\text{CvCO}}_{2}\text{=}r{\text{VCO}}_{2}\text{/}r\left(\text{S}{\text{. etCO}}_{2}\right)-{\text{CvCO}}_{2}$$

Hence: CO=nVCO2/n(S. etCO2)−CvCO2=rVCO2/r(S. etCO2)−CvCO2

$$\text{Finally: CO = Δ}{\text{VCO}}_{2}\text{/}\Delta \left({\text{S. etCO}}_{2}\right).$$

**Figure 2:**
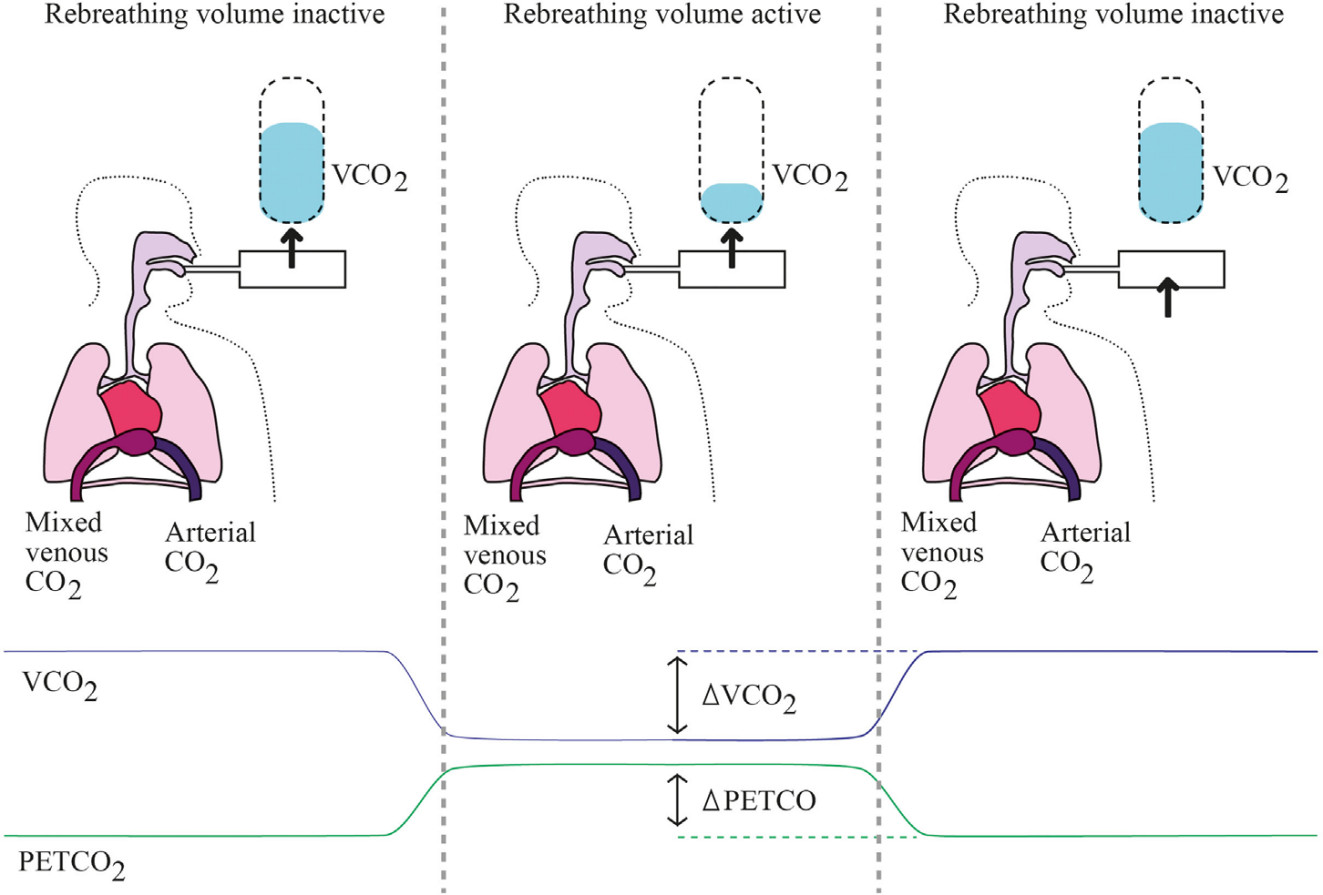
Partial rebreathing principles. Left panel represents baseline state, where the rebreathing valve is off and every parameter is at baseline levels. Middle panel represents early rebreathing time, when the valve is put; there is a decrease of VCO_2_ with simultaneous rise in PaCO_2_ and PETCO_2_. Right panel represents late rebreathing time when valve is off again and all parameters return to baseline levels, while mixed venous PCO2 has varied.

etCO_2_ can be measured in exhaled gas with a sealed facial mask. This partial CO_2_-rebreathing method hence allows measuring CO without the need of intravascular monitoring devices. Practical use involves an extra loop of ventilatory circuit to create a transient partial CO_2_ rebreathing system (i.e., etCO_2_) (see Figure 2).

The NICO-sensor (Philips Respironics, Eindhoven, the Netherlands) and INNOCOR (Innovision ApS, Denmark) are based on these principles (38, 39). Several limitations surround this method: (a) the smallest variations in CO_2_ can lead to significant differences in CO measurements, i.e., the slightest leaks in facial mask can induce measurement bias, (b) changes in ventilation modify end-tidal CO_2_ requiring patient respiratory state to be steady, i.e., not applicable in ICU, and (c) differences in VCO_2_ and end-tidal CO_2_ only account for that part of the lung which is ventilated, hence, atelectasis or intrapulmonary shunts need to be adjusted for, which in an ICU setting can prove difficult when patients present with several lung diseases (40–42). The two most recent validation articles published were small-sample studies in which this method was compared with thermodilution. Both failed to prove the equivalence between the two methods (43, 44).

A very recent retrospective study, in more than 12,000 patients who underwent right heart catheterization but were not necessarily hospitalized in ICU, found between thermodilution and an oxygen-uptake-based Fick method, an acceptable systematic bias of 0.4% but poor limits of agreement from −1.31 to +1.27 l/min; and a difference of more than 20% between measured CO in 40% of patients (45).

Hence, partial CO_2_-rebreathing is still hard to routinely use in ICU but fields of development include better rebreather-face interface to avoid leaks (i.e., masks) and correction algorithms which may take into account changes in end-tidal CO_2_, all the more in ICU setting. Indeed, this latter concern seems particularly difficult to address, as acute respiratory disease (including acute pulmonary edema, pneumonia and chronic obstructive pulmonary disease exacerbation) represents the most prevalent cause of admission in ICU.

## Pulse Wave Transit Time (PWTT)

**Figure 3:**
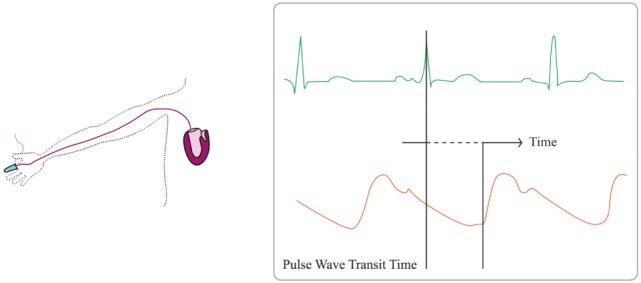
Pulse wave transit time principles. Pulse wave transit time is based on the delay of the generation of a stroke volume (orange line) after the generation of the R-wave on the electrocardiogram (green line).

Pulse wave transit time is the time required for a pulse pressure wave to travel between two points. It can be estimated from the time interval between the development of the R-wave on the electrocardiogram and its peripheral detection (see Figure 3). Approximating systemic blood circulation to a three-component Windkessel circuit (integrating aortic characteristic impedance, arterial compliance, and systemic vascular resistance) and neglecting vascular inertance, blood pressure can be associated with blood flow hence CO in a complex non-linear function (46, 47). PWTT is then considered inversely correlated with the SV (48). With increasing blood pressure, increasing arterial distending pressure and decreasing arterial compliance, pulse-wave velocity increases and PWTT shortens. Hence, PWTT was suggested as a surrogate measure of blood pressure changes. Given a known and fixed distance between the heart and the extremity on which the measurement is made, PWTT can be computed using the following Bramwill and Hill formula (49):

PWV=dP.V/ρ.dV,

where PWV = pulse wave velocity; ρ = density of blood; *V* = initial vessel volume; d*P* = the change in pressure; and d*V* = the change in vessel volume.

One product uses this technology (EsCCO, Nihon Kohden, Japan). Continuous CO is estimated with a multimodal algorithm PWV and using patients’ characteristics and several measurements such as pulse oximeter waveform, non-invasively measured blood pressure and electrocardiogram. The final formula is given by:

SV =K×(α×PWTT+β),

where the unique variable is PWTT then inversely proportional to velocity. Other determinants are α = −0.3, experimental proportional constant according to unpublished preliminary data and *K* and β are individual CFs based on physical profile (age, weight, height) and the initial measurement of the pulse pressure. Interestingly, initial CO was estimated only by this non-invasive patient information calibration (50). Even if later refined by an automated exclusion algorithm, several concerns were raised as to its accuracy in ICU setting (51–55). Indeed, although systematic bias was acceptable with 0.13 l/min, limits of agreement were poor (between −2.13 and 2.39 l/min) (51). Limitations include vasoconstriction, cold extremities and arrhythmias all of which induce bias in measurements. Moreover, while calibration with invasive means seems to enhance the trueness of this device; there is uncertainty as to its stability (51). Finally, catecholamines infusions are a limitation to the use of plethysmographic-variability-based indices in critically ill patients (56, 57).

While EsCCO has not been quite validated in ICU, devices using pulse wave contour analysis, working quite closely to pulse wave velocity analysis are more promising. EsCCO suffers mainly from initial individual calibration issues, which are reduced to a crude algorithm aggregating a few variables which may not be sufficient to account for the wide variability of patients presenting in ICU. Indeed, the two main issues are (i) the heterogeneity of patients’ profiles, for which an overall algorithm may be statistically true for most but containing an inherent percentage error, making individual prediction hard to assess and (ii) the interpatient variability in the course of his treatment and care in ICU (accounting for volemia, vasoconstriction or vasodilation, catecholamine use and arrhythmia, to name a few).

## Ultrasonic Methods

Product of aortic blood flow velocity and area of a section of the aorta equals to the CO measured in the aorta. Blood flow velocity can be measured using ultrasound and Doppler effect

**Figure 4:**
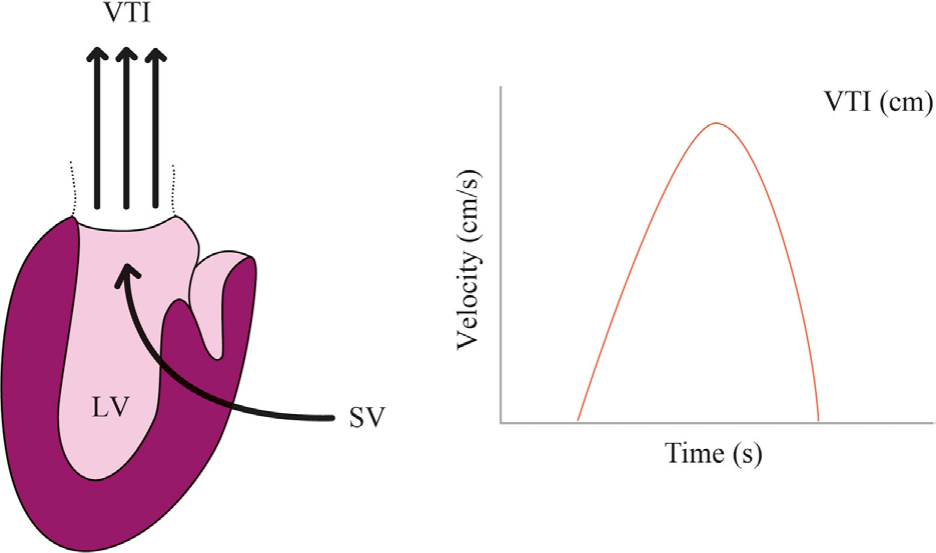
Echocardiographic monitoring. Aortic flow velocity time integral (VTI) multiplied by the cross-sectional area (CSA) allows to compute stroke volume (SV) ejected by the left ventricle (LV). Heart rate (HR) then allows to compute cardiac output (CO) = VTI × CSA × HR.

SV=VTI.CSA,

where VTI = aortic flow velocity time integral and CSA = aortic cross-sectional area. Hence, a non-invasive measurement method would require a device continuously measuring aortic blood flow, in a fixed manner (see Figure 4). This method is used in the ultrasonic cardiac output-monitoring (USCOM) device. USCOM requires the precalculation of the aortic valve area based on patient’s age and weight. Moreover, ICU setting seems to be inadequate for using USCOM (58–60). Limitations include (i) the difficulty of keeping the USCOM Doppler probe in a steady position on a critically ill patient, (ii) the lack of echogenicity in patients who underwent cardiac surgery (61), and (iii) the reliability of the valve area estimation based on age and weight tends to decrease with population age (62, 63).

A few articles highlight the feasibility of using USCOM in ICU, with a systematic bias of −0.36 l/min however limits of agreement were poor ranging from −2.34 to 1.62 l/min and the reported percentage error (29%) seemed too high for daily use (64).

To put it in a nutshell, although point-of-care ultrasonic evaluation of CO is widely used in ICU, continuous echocardiographic monitoring of CO by USCOM remains largely debated. Indeed, a high percentage error, either due to errors in valve area estimation or probe displacement, make it hard to routinely apply. However, initial calibration on actual echocardiographic assessment of the valve area and regular signal-quality checks may improve this technique.

## Inductance Thoracocardiography

This method allows the computation of ventricular volume curves from ECG-triggered ensemble respiratory waveform of an inductive plethysmographic transducer. The latter is placed on the thorax by surrounding with a belt. Impedance varies according to respiration and cardiac ejection. Because the transducer is positioned in front of the heart, heartbeat-related ventricular volume variations are detected and adjusting the signal on respiratory-related impedance signal allows computing specific cardiac changes. The only device using this technology is Respitrace (Noninvasive Monitoring Systems, Miami, FL, USA) (65).

Main limitation of this method resides in the fact that it only detects relative variations in cardiac volumes (66, 67), hence, at least one calibration per patient is required to get an absolute value (68). Moreover, if thoracic compliance is very low, cardiac volume variations can be undetectable. Finally, although the method was published at the end of the 90s, only a few publications have since been written by a few authors only, making external validation difficult to assess. In 2017, inductance thoracocardiography seem like it fell out of clinical practice, maybe to the exception of a few experimental settings.

## Discussion

The need for a non-invasive, true and precise CO measurement in the ICU is, as of yet, still unsatisfied (69, 70), despite acceptable results on other settings. As recent reviews demonstrated, overall, validation articles available in the field of non-invasive hemodynamic monitoring showed too large heterogeneity and devices, insufficient levels of agreement. Thus, further research may be warranted in the field, as hemodynamic monitoring is bound to be less and less invasive in the future.

Extensive reviewing of published data on diagnostic performance of monitoring devices, be they invasive or not, shows heterogeneity in reporting of performance. Specifically, *accuracy*, i.e., how close a single measurement value is to the true value of the measurand can never be numerically assessed. Indeed, the true value of the measurand can only be approximated by a reference method or, when available, a gold-standard. Theoretically, if someone could repeat the measurement an infinite number of times to estimate the same measurand value, the only difference between the averaged observed value and the true value would equal the systematic measurement error (i.e., systematic bias qualifying the trueness). Statistical analyses are aimed for adjusting for such bias, however, most methods derive from population-based algorithms, hence do not account for individual variability. Therefore, non-invasive devices are characterized by acceptable mean interpatient bias but poor individual calibration. *Precision*, as defined by metrological standards, represents the repeatability and reproducibility of the method, i.e., the degree to which repeated measurements using the same method to estimate the same measurand value, produce the same observed value. Inherently, it relates to random measurement error (as opposed as systematic measurement error represented by the bias). As such, most publications do not specify precision but rather publish the standard deviation of the bias in the cohort, i.e., interpatient bias. A higher precision allows for fewer measurements in order to have an estimation of the measurand. Hence, *precision* has a direct practical impact on the usability of devices, especially in the step time response of the device. Indeed, very few articles describe how many measurements were taken to obtain a value, and similarly, manufacturers do not always specify how many measurements are necessary to be within acceptable error limits. In practice, non-invasive devices present the obvious advantage of allowing repeated measures to obtain more accurate value, given they would be adequately calibrated. However, if a given device takes too long to estimate a measurand, its usefulness may be challenged, however accurate it can be.

Hence, the risk of misdiagnosis or delay to diagnosis from an insufficiently accurate non-invasive device remains real. Indeed, they represent the counterparts of invasive device-related complications, be they infections or hemorrhages. Consequently, properly assessing the need for invasive monitoring remains a clinical challenge in ICU, to which, the only acceptable solution would be equally efficient non-invasive devices.

Interestingly, obtaining the true value of a measurand would not necessarily be the most important feature that one might require from a hemodynamic monitoring device. Indeed, ability to observe variations in hemodynamics is equally important, if not more; implying fast step-time response and precision. Observing the decrease in CO may be as useful as knowing this exact value. In the end, the difference between trueness and precision may be analogous to that of diagnosis or monitoring.

## Conclusion

Non-invasive monitoring has evolved in the past few years, seeing the appearance of promising new devices. Further developments may be warranted to validate their use and increase their metrologic performance in ICU. Even though some have successfully deployed such device, the need for a non-invasive, true and precise CO measurement in ICU is, as of yet, still unsatisfied.

## Author Contributions

LN and PS contributed equally to the manuscript.

## Conflict of Interest Statement

LN has no conflict of interest regarding this publication. PS perceived consulting fees from Edwards and Cheetah Medical prior to 2000.
